# p16 overexpression in malignant and premalignant lesions of the oral and esophageal mucosa following allogeneic hematopoietic stem cell transplantation

**DOI:** 10.1186/1758-3284-4-38

**Published:** 2012-06-23

**Authors:** Yasumasa Kakei, Masaya Akashi, Hideki Komatsubara, Tsutomu Minamikawa, Takahide Komori

**Affiliations:** 1 Department of Oral and Maxillofacial Surgery, Institute of Clinical Medicine, Kobe University Graduate School of Medicine, Kusunoki-cho 7-5-1, Chuo-ku, Kobe 650-0017, Japan; 2 Department of Oral and Maxillofacial Surgery, SHAKAI-HOKEN, Kobe-central Hospital, Souyama-cho 2-1-1, Kita-ku, Kobe 651-1145, Japan

**Keywords:** Squamous cell carcinoma, Dysplasia, Acanthosis, Intraepithelial neoplasia, Hematopoietic stem cell transplantation, Human papillomavirus, p16

## Abstract

**Objectives:**

Secondary malignancy in the oral mucosa is recognized as one of the most serious complications in patients who received allogenic hematopoietic stem cell transplantation (HSCT). However, potential risk factors associated with carcinogenesis after HSCT that have been reported remain elusive. We experienced a rare case of secondary malignancies of the oral and esophageal mucosa and analyzed the expression of tumor suppressor gene product p16.

**Case report:**

A 35-year-old male had malignant lesions of the oral and esophageal mucosa two years after HSCT. Partial maxillectomy and endoscopic submucosal dissection were performed. Immunohistochemical analyses revealed that the tumor cells of malignant and premalignant lesions of the oral cavity and esophagus but not keratosis were positive for p16.

**Conclusions:**

Pathological examinations with p16 immunohistochemistry may contribute to an early diagnosis of secondary malignancy after HSCT.

## Background

Allogeneic hematopoietic stem cell transplantation (HSCT) is an increasingly therapy for hematological malignancies in recent years. Despite significant improvement of survival rate, the development of secondary malignancy has been recognized as one of the most serious long-term complications after HSCT. Malignancies occurring after HSCT can be classified into three categories: hematological malignancy and lymphoproliferative disorder, and solid tumor [1]. Potential risk factors associated with the development of secondary malignancy after HSCT that have been reported include cytotoxic effects of chemotherapeutic agents or iarradiation, chronic graft-versus-host diseases (GVHD) related inflammation, immunosuppression for GVHD propylaxis, viral infection and chronic stimulation as a result of viral antigens [2,3].

Secondary solid tumor seems to be less common, but the incidence increases significantly over time in long-term survivors of allogenic HSCT [4]. Oral squamous cell carcinoma (SCC) is the most common secondary solid tumor [1,4,5]. It is considered that the presence of chronic GVHD in oral mucosa has a significant role in the pathogenesis of oral cancer after allogeneic HSCT [6]. The frequent evaluations for oral chronic GVHD by oral medicine specialists and the appropriate pathological examinations with useful biomarkers may contribute to an early diagnosis of oral malignancy.

The p16 protein has been identified as a tumor suppressor that functions by inhibiting the cyclin-dependent kinases 4 and 6. Overexpression of p16 protein has been reported repeatedly in HPV-associated cancers. p16 immunohistochemistry also has been described as a potential marker to recognize the presence of dysplasia of oral cavity [7].

Here, we report a rare case of oral and esophageal malignancy after allogeneic HSCT. An immunohistochemical analysis of p16 in malignant, premalignant, and nonmalignant lesions was performed.

## Case report

### Clinical history

**Figure 1:**
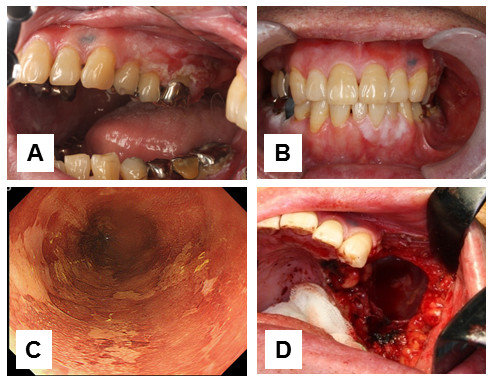
**Erosive mass in the upper gingiva (A).** White patches in the lower gingiva **(B)**. Lesions unstained with lugol’s solution in the esophagus **(C)**. Partial maxillectoy **(D)**.

A 35-year-old male diagnosed with malignant lymphoma of the small intestine was treated with surgery and chemoradiotherapy. Subsequently, he had a complete response after autologous peripheral blood stem cell transplantation (PBSCT). Eight years after PBSCT, he was diagnosed with treatment-related acute myeloid leukemia and underwent allogeneic HSCT stem cell from a fully human leukocyte antigen–matched sibling. He received cyclophosphamide and total body irradiation for conditioning. Short-course methotrexate and cyclosporine A (CSA) were used for GVHD prophylaxis. A month after HSCT, he developed interstitial pneumonia due to acute GVHD. He had lichenoid lesions in the oral mucosa, keratoconjunctivitis sicca, and bronchiolitis obliterans three months after HSCT. Oral biopsies revealed the diagnosis chronic GVHD and showed acanthosis of the oral epithelium. He was treated with CSA and prednisone. Two years after HSCT, he presented with an erosive mass in the upper gingiva and adherent white patches in the lower gingiva (Figure 1A, B). The diagnosis of oral SCC in the upper gingiva and low grade dysplasia in the lower gingiva was confirmed by biopsy. Esophagogastroduodenoscopy (EGD) that was routinely performed for oral malignancy patients in our hospital revealed high grade intraepithelial neoplasia in the esophagus (Figure 1C). The malignant and premalignant lesions of oral cavity were surgically removed by partial maxillectomy (Figure 1D) and CO2 laser abration. Two month after the operation, high grade intraepithelial neoplasia in the esophagus was treated with endoscopic submucosal dissection (ESD). Two years after the operation, the patient could not be followed-up in our hospital, because his lung function worsened due to chronic GVHD.

### Pathological findings

Immunohistochemical staining was performed using antibodies against p16 (Epitomics, Burlingame, USA), p53 and Bcl-2 (Dako, Glostrup, Denmark). The procedure was done according to the manufacturer’s instructions. Well-characterized samples of nonmalignant, premalignant and malignant mucosa for immunohistochemistry were selected from the biopsy specimens.

**Figure 2:**
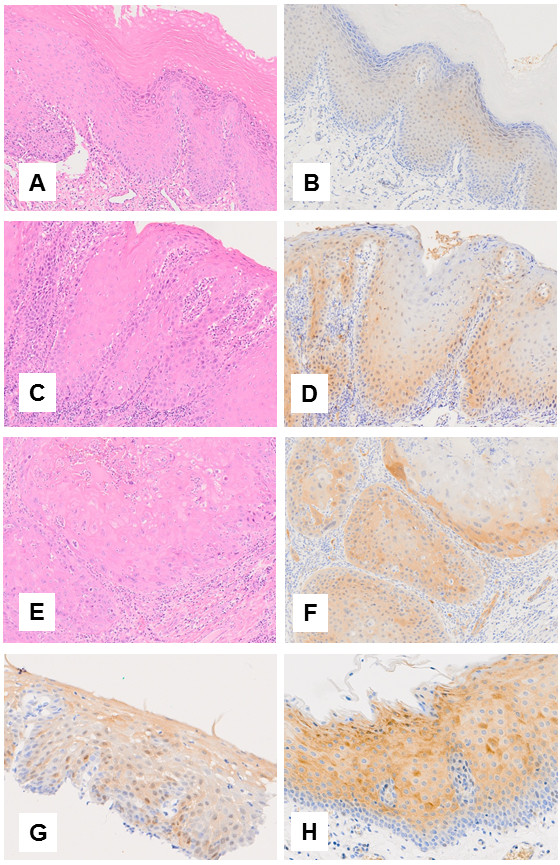
**Immunohistochemical analyses for the expression of p16.** Acanthosis **(A, B)**, oral epithelial dysplasia **(C, D)** and oral squamous cell carcinoma **(E, F)** (H&E × 20) and (DAB peroxidase × 20). High grade intraepithelial neoplasia **(G)**. The p16 positive control (lung adenocarcinoma) **(H)**.

The basal and suprabasal cells’ nuclei of the acanthotic, dysplastic and malignant lesions were positive for p53 (data not shown). The lymphocytes below the epithelium and around SCC but not tumor cells were positive for Bcl-2 (data not shown). The tumor cells of malignant and premalignant lesions of the oral cavity and esophagus but not acanthosis were positive for p16 (Figure 2).

## Discussion and Conclusions

Among the late effects after HSCT, secondary solid tumors are generally less frequent than hematological malignant disorders, but their incidence increases over time. Skin and mucosal neoplasms account for approximately one-third all secondary solid tumors in HSCT patients, with oral squamous cell carcinoma representing 50 % of these cases [4,5,8]. However, details of the clinical aspects of oral malignancies following HSCT have been only described in several case reports [9,10]. Although possible mechanisms have been previously reported, the immunohistochemical analyses for secondary malignancies after GVHD are few.

GVHD is a common complication in patients who are treated with allogeneic HSCT. It is considered that the presence of chronic GVHD in oral mucosa has a significant role in the pathogenesis of oral cancer after allogeneic HSCT [2]. Lichen planus-like lesions that include hyperkeratotic striae and patches combined with ulcerations are the most distinctive oral change of GVHD. Differential diagnosis of oral dysplasia that may undergo malignant transformation is sometimes difficult in these patients.

The presence of p53 tumor suppressor gene mutations is common molecular defects in human malignancies, including oral SCC. p53 mutations may be related to prolonged exposure to immunosuppressive drugs (e.g. CSA) [11]. However, overexpression of the mutated p53 protein, probably related to the inflammatory infiltrate, was detected in epithelium of oral lichen planus without dysplasia [12]. In our case, p53 expression was observed in the non-malignant oral mucosa as well as malignant lesions. Bcl-2 is one of the most studied anti-apoptotic oncoprotein. Some have shown an increase in Bcl-2 expression in dysplastic and malignant lesions of the oral cavity [13,14]. In our case, only the infiltrating lymphocytes under the epithelium and around the tumors were positive for Bcl-2. Therefore, p53 and Bcl-2 immunostaining is not considered to be appropriate for the differential diagnosis of oral dysplasia.

Although remarkable correlation between HPV detection and p16 overexpression in oropharyngeal carcinomas has been reported, p16 overexpression was not a reliable predictor of HPV infection in young patients with tongue SCC [15]. One study showed that all oral SCC after HSCT demonstrated pappillomatous aspects (a sign of HPV infection) as well as chronic GVHD features (dense subepithelial lymphoid infiltrate) [16]. In other report, HPV was not associated with oral carcinogenesis after HSCT [17]. The association of HPV infection with esophageal intraepithelial neoplasia is also controversial. However, it was previously reported that the only HPV-positive cases showed strong p16 staining, compared with HPV-negative cases in esophageal squamous intraepithelial neoplasia [18]. In our case, p16 overexpression was detected only in malignant and premalignant lesions but not keratotic lesions. Pathological examinations with p16 immunohistochemistry may contribute to an early diagnosis of oral and esophageal malignancy in immunocompromised patients after HSCT.

## Consent

Written informed consent was obtained from the patients for publication of any accompanying images.

## Abbreviations

Bcl-2, B-cell lymphoma 2.

## Competing interests

The authors declare that they have no competing interests.

## Authors’ contributions

YK participated in the surgery and data collection. MA participated in the surgery, designed and drafted the manuscript. HK was a physician in charge before the surgery. TM performed surgery. TK revised the article for important intellectual content. All authors read and approved the final manuscript.
